# Myelin Protection by Ursolic Acid in Cuprizone-Induced Demyelination in Mice

**DOI:** 10.22037/ijpr.2019.112181.13582

**Published:** 2019

**Authors:** Fatemeh Honarvar, Vida Hojati, Nuredin Bakhtiari, Gholamhassan Vaezi, Mohammad Javan

**Affiliations:** a *Department of Biology, Damghan Branch, Islamic Azad University, Damghan, Iran. *; b *Department of Biochemistry, Faculty of Biological Science, North_Tehran Branch, Tehran, Iran. *; c *Department of Physiology, Faculty of Medical Sciences, Tarbiat Modares University, Tehran, Iran. *; d *Department Brain and Cognitive Sciences, Cell Science Research Center, Royan Institute for Stem Cell Biology and Technology, ACECR, Tehran, Iran.*

**Keywords:** Multiple sclerosis, Myelin protection, Myelination, Ursolic acid, Corpus callosum, Mouse

## Abstract

Neuronal survival in multiple sclerosis (MS) and other demyelinating diseases depends on the preservation of myelin and remyelination of axons. Myelin protection is the main purpose to decrease myelin damage in the central nervous system (CNS). Ursolic acid (UA) as a natural product in apple is suggested to protect neural cells. This study is the first to demonstrate an effect for UA on CNS myelin loss induced by cuprizone toxin. In the current study, we hypothesized that daily treatment with UA in drinking water (1 mg/mL) prevents myelin damage by 6 weeks administration of CPZ in mice pellet which lead to corpus callosum axonal demyelination. We assessed the myelin content and the number of myelinating cells in corpus callosum by FluoroMyelin and luxol fast blue staining as well as by immunostaining against MBP and Olig2. Our finding indicated that UA could decrease the extent of demyelination area and enhanced myelin stain intensity within CC and protected oligodendrocyte lineage cells against cuprizone toxin. We could conclude that myelinated structures could be protected by UA in corpus callosum, which provide favorable evidence for the possibility of application of UA in demyelinating diseases and traumatic injuries.

## Introduction

Myelin, a protective coating around the nerve fibers facilitates action potential conduction in the nervous system. It is involved in salutatory conduction of neural impulses and accelerates the conduction velocity by 20-100-fold compared to non-myelinated axons of the same diameter (1). Glial cells, called oligodendrocytes (OLs), are the myelinating cells in the central nervous system (CNS); however in peripheral nervous system (PNS), myelin is formed by Schwann cells. Among neurological disorders, multiple sclerosis (MS) disrupts the flow of electrical signal through demyelination and the destructions of insulating covers of nerve cells (2). Following myelin damage, endogenous remyelination will be activated through differentiation of oligodendrocyte progenitor cells (OPCs) as well as the neural stem cells (NSCs) to OLs, although it may be insufficient (3). Therefore, an imbalance between demyelination and remyelination processes occurs in MS. Accordingly, OLs protection may be considered as the primary therapeutic target in MS patients, although several research labs also focus on strategies to enhance myelin repair (4-12).

Axonal survival depends on myelin therefore myelin protection and remyelination of axons contributes to axonal survival and prevent transition to progressive phase of MS (13, 14). Most of the current treatments of MS target the immune system by reducing its activity or blocking entry of immune cells into the CNS; however immune-mediated damage to the myelin sheaths surrounding the axons gradually proceed and leads to axonal damage. (15). Furthermore, OLs consume high energy for production of extensive amounts of lipid-rich myelin membrane; apparently remyelination creates very high metabolic demand and may lead to metabolic deficiencies and cellular dysfunction as well as limited remyelination (16). On the other hand, demyelination also leads to increased metabolic demand in axons and consequently axonal damage happens. Unhealthy axons may no longer be receptive to remyelination when neurodegeneration proceeds. Therefore, neuroprotective strategies that protect myelin against the responsible mechanisms can improve OLs and axons survival and by decreasing axonal degeneration may reduce disease protection. 

Ursolic acid (3β-Hydroxy-12-ursen-28-ic acid) is a natural pentacyclic terpenoid (17) which is known as an anti-inflammatory agent (18) with anti-oxidant effects (19, 20). Ding *et al*. (21) demonstrated that UA activated the Nrf2 signaling pathway, as a mechanism of brain protection and experimental traumatic brain injury in mice. Therefore, protective effects in neurodegeneration diseases are expected. UA also inhibited apoptosis signaling by reducing caspase-3 and caspase-9 in mRNA and protein levels (20). UA also inhibited β-Amyloid (Aβ)-induced caspase-3 activity, attenuated Aβ -induced apoptosis, and protected PC12 cells against Aβ neurotoxicity in a dose-dependent manner, that highlight the neuroprotective effect of UA (22). In brain diseases, cessation of the malondialdehyde (MDA) levels, increasing of reduced glutathione (GSH), recovery of the suppressed superoxide dismutase (SOD), and catalase (CAT) activities following UA administration suggest a protective effect in response to oxidative stress. Furthermore, UA promoted the regeneration of injured myelin sheaths in PNS (23), although no reports have explored its effect on CNS myelin.

In the current study, we hypothesized that UA may protect myelin in CNS in the context of demyelinating conditions. To examine the protective effects of UA, mice received daily treatment of UA in drinking water when fed with cuprizone (CPZ) to induce demyelination in corpus callosum (CC). We showed myelin protection using different myelin staining methods including LFB, FluoroMyelin and immunohistofluorescence studies.

## Experimental

*Animals*

Adult male C57BL/6 mice weighing 20-25 g (8–10 weeks) were purchased from Pasteur Institute (Karaj, Iran). The animals were maintained three per cage under 12 h light/dark cycles under controlled temperature (23 ± 2 °C). Food and water were freely available. All experiment procedures were designed based on international guidelines for animal studies and approved by the ethical committee for animal research, Tarbiat Modares University, Tehran. Efforts were made to minimize both the number of animals used and their suffering.

*Interventions*

For induction of demyelination, the male C57BL/6 mice were fed with a diet containing 0.2% cuprizone (oxalic acid bis (cyclohexylidene hydrazide); Sigma-Aldrich Inc., St. Louis, MO, USA) mixed into their normal chow for 6 weeks. The mice were divided into three groups including control with normal water, cuprizone diet with normal water, and cuprizone diet with water containing UA (1 mg/mL) (Enzo Life Sciences). The animals’ brains were collected at 6 weeks post cuprizone. The animal’s weight and food intake were recorded every other day. 

*Y-maze behavioral test*

Y-maze test was performed to confirm the presence of behavioral impairment due to demyelination in their brains. Spontaneous alternations within the Y-maze were calculated prior to induction of demyelination and 6 weeks after feeding with cuprizone diet. The Y-maze arms were labeled as A, B, and C. The number of overlapping entrance sequences (e.g., ABC, BCA) was defined as the number of spontaneous alternations. The percentage of alternations was calculated as follows.

$\mathrm{Alternations}\left(\%\right)=\frac{\mathrm{Number of alternations}}{\left(\mathrm{Total number of arm entries}-2\right)}\mathrm{*100}$

*Processing and sectioning of brain tissues*

The animals were perfused with phosphate buffer saline. The brains were removed and the hemispheres were dissected. The right hemispheres were post-fixed in 4% paraformaldehyde 24 h, then in sucrose 30% (2-3 days), and embedded in optimal cutting temperature (OCT) for long term preservation in -80 °C. Ten micrometer thick sections were cut using a cryostat (Histoline, Italy) and mounted on superfrost plus slides for later evaluations. The sections were stored at -20 °C until final staining. 

*Luxol Fast Blue and Cresyl Fast Violet staining*

To evaluate the myelination intensity and the extent of demyelination in corpus callosum, the sections with 10-μm thick were selected for myelin staining. For FluoroMyelin (FM) staining, the sections were incubated with red FM (Invitrogen F34652) for 20 min at room temperature. FM solution were prepared by diluting FM 1:300 in PBS. 

For Luxol fast blue (LFB) staining the sections were stained with 0.1% LFB solution (British Drug House, London, UK) at 60 °C for 1 h. Adequate contrast was made by rapid immersion of preparations in 0.05% lithium carbonate followed by several changes of 70% alcohol. The sections were washed with distilled water, and then counterstained with 0.1% Cresyl Fast Violet (Merck, Germany) for 4 min. After washing with distilled water, the sections were dehydrated in a graded series of alcohols, cleared in xylene, coverslipped, and screened for demyelination and subsequent quantitative analysis. For each section, the extent of demyelination was assessed by using Image J software as the percentage of whole corpus callosum. The extent of demyelination was averaged for 8 sections obtained from each animal and then averaged for each animal group (n = 3 for each data point).

*Immunostaining*

To perform the immunofluorescence staining against myelin antigens, the sections were washed three times with PBS containing 0.05% Tween 20, then incubated in blocking solution for 1 h at room temperature. Blocking solution consisted of 0.1% Triton X-100 (Sigma-Aldrich, T8532), 1% bovine serum albumin, and 5% normal serum in PBS. Then, the samples were incubated at 4 °C for overnight with primary antibodies against Olig_2_ and MBP. Afterward, the sections were washed three times with PBS and incubated with corresponding secondary antibody for 1 h at room temperature. The list of antibodies used in this experiment is provided in Table 1. To counterstain and visualize cell nuclei, the samples were treated with 4´,6-diamidino-2-phenylindole (DAPI; Sigma-Aldrich; D-8417), and again washed with PBS for three times. The sections were coverslipped and then the images were obtained using an Olympus BX 51 Microscope and DP-72 camera for consequent analysis. The Analysis was performed on 8 to 12 sections, which were randomly chosen per animal. Areas corresponding to corpus callosum in one-half of the brain were analyzed for myelination intensity.

*Data Analysis*

Each animal group included 3-6 mice. Statistical analyses were conducted using GraphPad Prism (GraphPad Software, La Jolla, CA, USA). One-way analysis of variance ANOVA, followed by Tukey post-test was used for comparing the experimental groups. The results were expressed as mean ± SEM. The differences were considered significant when *p*-value was less than 0.05. 

## Results

*Ursolic acid effects on CPZ feeding and water drinking*

**Figure 1.**
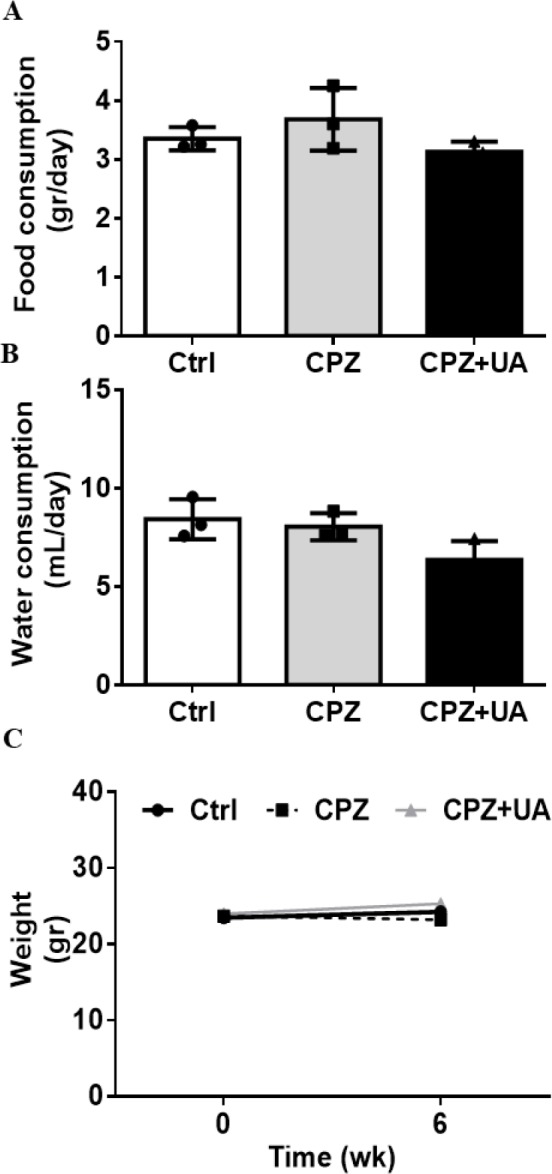
The rate of food (A) and water (B) consumptions and mice weight (C) during 6 weeks with and without cuprizone diet or UA

To identify the UA effect on the normal appetite of in CPZ-fed mice, we used UA as a dietary supplement in drinking water during 6 week after CPZ-exposure. The results indicated no significant difference between the food intake, the amount of drinking water, and the average animals’ weight after 6 weeks. (Figure 1). 

The same amount of food intake stands for the same amount of CPZ delivery to the UA treated and non-treated mice. Therefore, the changes in the myelination and lesion extent in these two groups will be due to the UA treatment.

*Ursolic acid improved the spatial recognition memory*

**Figure 2:**
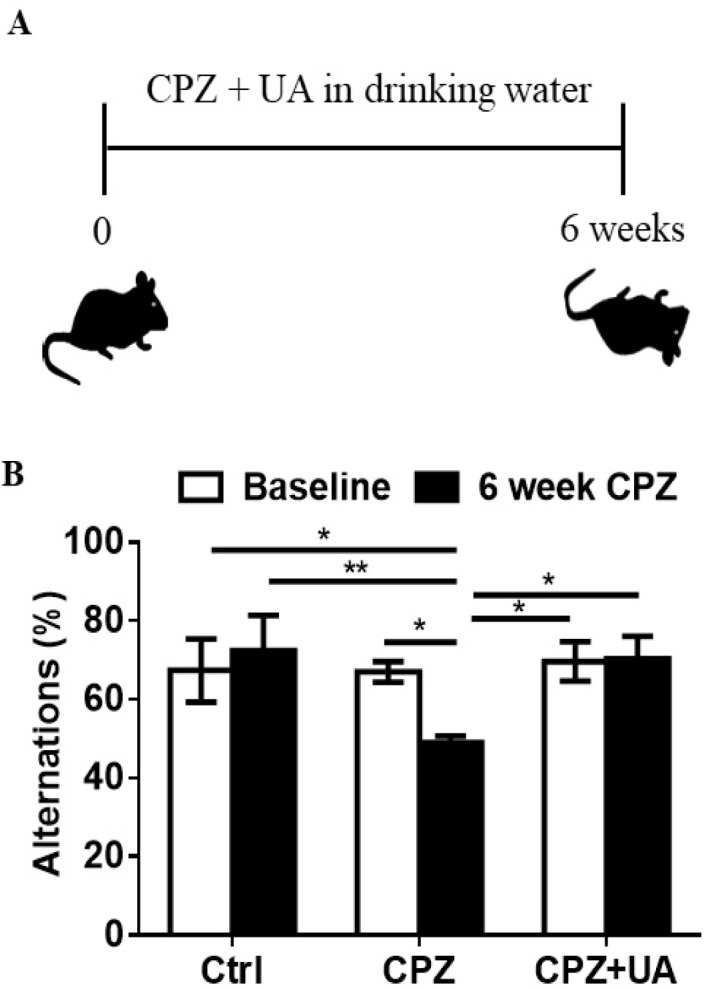
Effect of UA on memory performance in mice treated with cuprizone. (A) Schematic representation of the protocol of UA administration in drinking water (1 mg/mL). (B) Spontaneous alternations was assessed using the Y-maze test (n = 6). Ctrl: animals which received normal food and water, CPZ: animals which received normal water and cuprizone food, CPZ+UA: animals which received cuprizone food and UA in drinking water. *p < 0.05, **p < 0.01 compared to the CPZ group

CPZ administration for 6 weeks leads to memory impairment due to extensive demyelination within the brain. To investigate the protective effect of UA on the spatial working memory in CPZ-exposed mice, we used Y-maze as a simple short term memory test at baseline (week 0) and at week 6 post CPZ-exposure (Figure 2A). The results indicated that the mice exposed to CPZ for 6 weeks had fewer spontaneous alternations in Y-maze (*p < *0.05 compared to the baseline; *p *< 0.01 compared to the age-matched controls (Figure 2B). Therefore, UA could protect mice against the effect of CPZ-mediated demyelination on memory performance.

*Ursolic acid protected myelin in corpora callosa of CPZ-fed mice*

**Figure 3:**
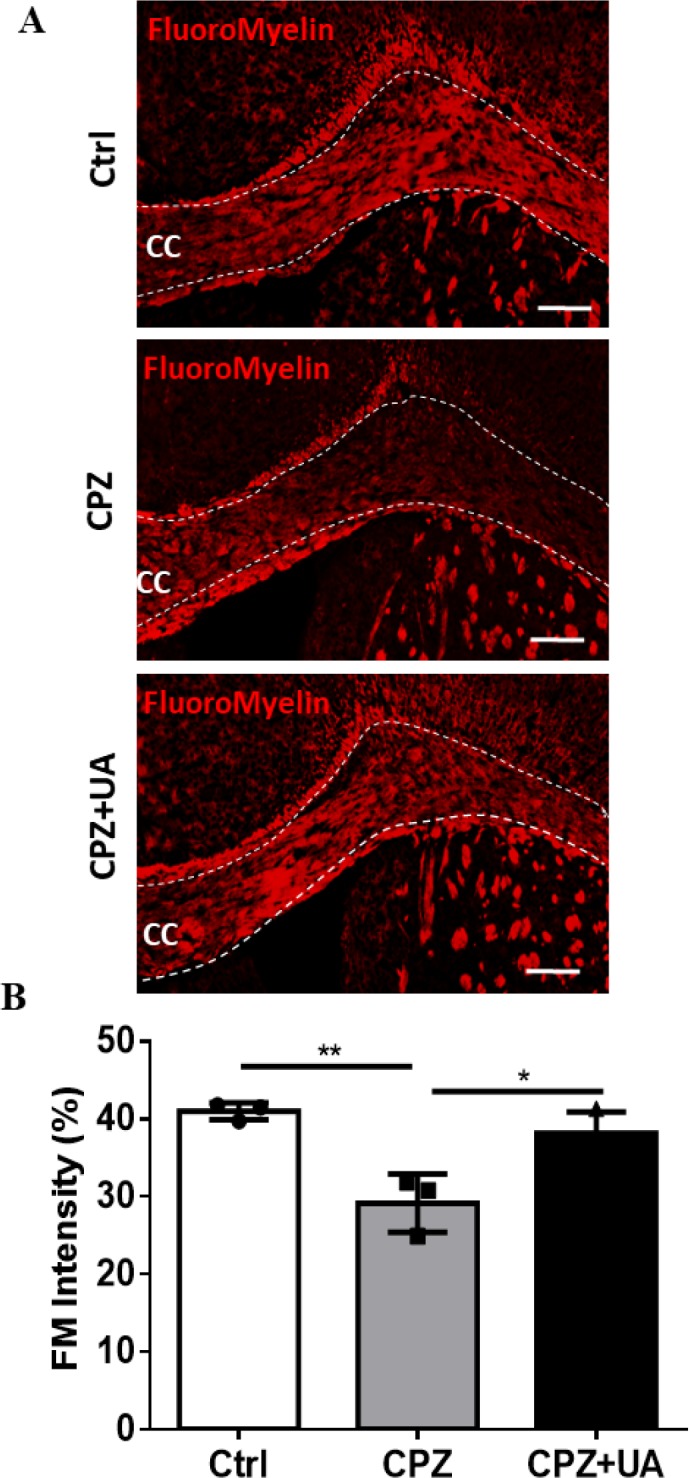
Effect of UA on the extent of demyelination area in corpus callosum. (A) FluoroMyelin (FM) staining against myelin for the control (Ctrl), cuprizone (CPZ), and cuprizone+UA (CPZ+UA) groups. (B) Quantitative analysis of the demyelination intensity in FM-stained sections. Ctrl: animals which received normal food and water, CPZ: animals which received normal water and cuprizone food, CPZ+UA: animals which received cuprizone food and UA in drinking water. *p < 0.05, **p < 0.01 compared to the CPZ group. *p < 0.05, **p < 0.01 compared to the CPZ group. CC: Corpus callosum. Scale bars: 100 μm

Demyelinated regions within the corpora callosa could be clearly discovered following FluoroMyelin (FM) staining as a selective fluorescent dye for myelin (Figure 3A). The loss of myelin was extensive at week 6 post CPZ-feeding when compared to the control (*p <*0.01). UA treatment for 6 weeks during the course of demyelination induction could reduce the amount of demyelinated area in corpus callosum when compared to CPZ group without treatment (*p < *0.05). There was no significant difference between this group and the control one with the same age which may imply the complete protection of myelin. All quantification and statistical comparisons are presented in Figure 3B.

**Figure 4:**
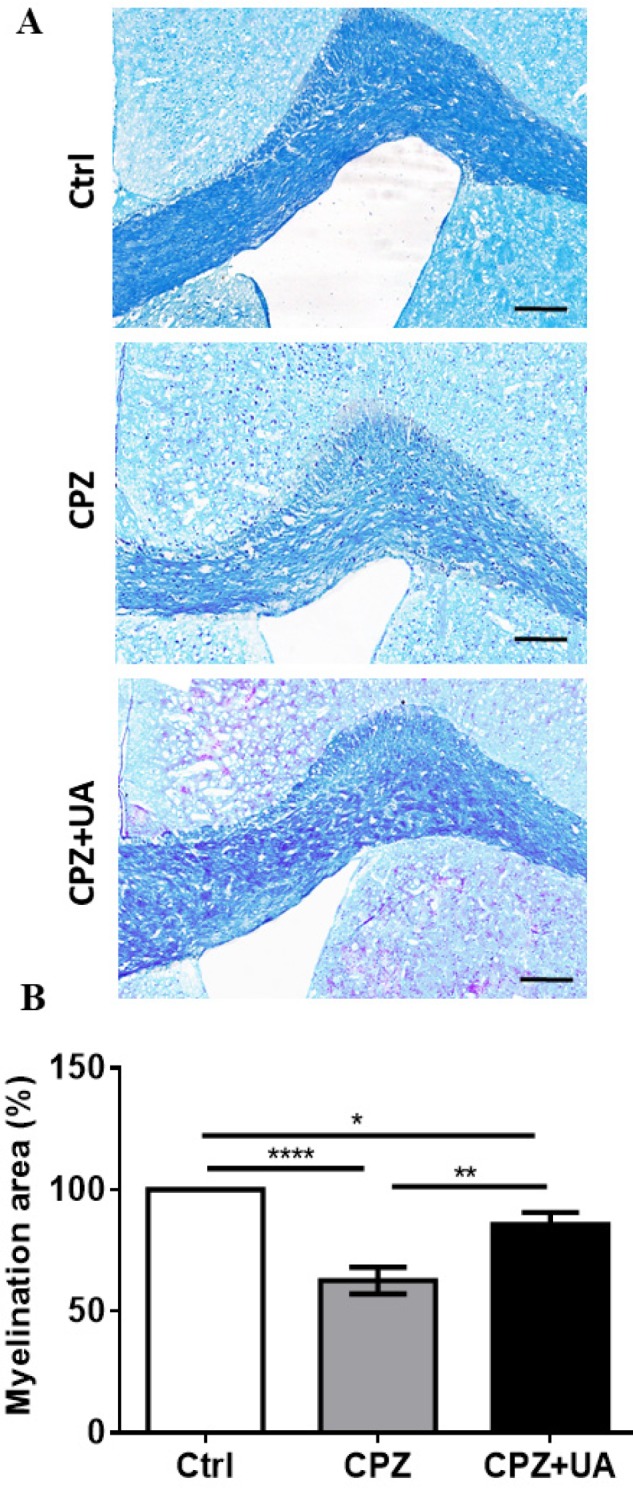
Effect of UA on the myelination intensity in cuprizone (CPZ)-induced demyelination model. Myelination was assessed at week 6 post CPZ. (A) Luxol Fast Blue (LFB) staining shows demyelination in corpus callosum (CC) of CPZ- fed mice and myelin preservation in the UA treated animals

To confirm the difference in the extent of demyelination within the corpus callosum, luxol fast blue (LFB) staining was performed at the end of 6 weeks CPZ feeding. Figure 4A shows the representative micrographs with notable reduced demyelination in UA-treated animals. Statistical comparison of animal groups showed a significant increase in the myelination intensity in CPZ fed animals treated with UA in drinking water when compared to the CPZ group (*p < *0.01, Figure 4B) but still lower than the control group (*p *< 0.05). 

Myelination intensity in the control group of the same age was considered as 100% myelination.

Mature OLs and fully myelination OLs express MBP as key protein in myelin compaction. 

**Figure 5:**
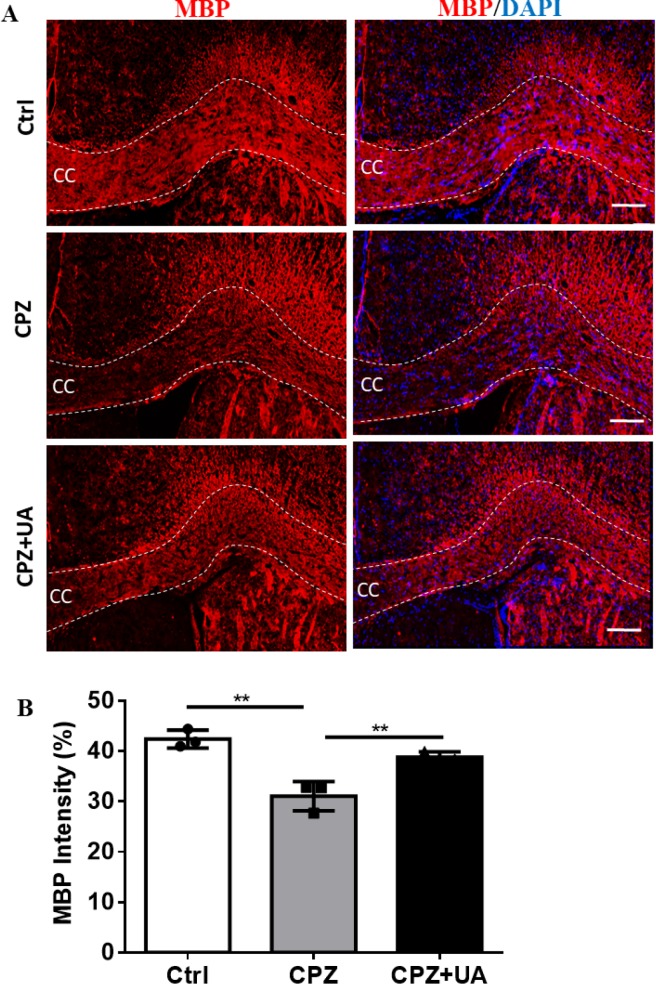
Determining the myelination levels by staining against myelin basic protein (MBP) as a myelin marker in corpus callosum (CC). (A) Immunofluorescence staining against MBP to evaluate the myelination level at CC. (B) Quantitative analysis of MBP-stained sections shows protective effect of UA. Ctrl: animals which received normal food and water, CPZ: animals which received normal water and cuprizone food, CPZ+UA: animals which received cuprizone food and UA. **p < 0.01 compared to CPZ group, Scale bars: 100 µm, n = 3

Therefore, myelin staining using specific antibodies provides a more reliable method for detecting the changes in myelination. Immunohistofluorescent study was used to assess the myelin levels in corpus callosum of the animal groups (Figure 5A). 

Staining against MBP showed that UA preserved the myelin compared to CPZ-exposure. Despite the marked reduction in myelin and MBP staining in the corpus callosum of CPZ-fed mice (*p *< 0.01), the level of myelin in CPZ+UA group was significantly higher than that in the control group (*p *< 0.01, Figure 5B).

*Higher number of oligodendrocyte lineage cells in ursolic acid treated animals*

**Figure 6:**
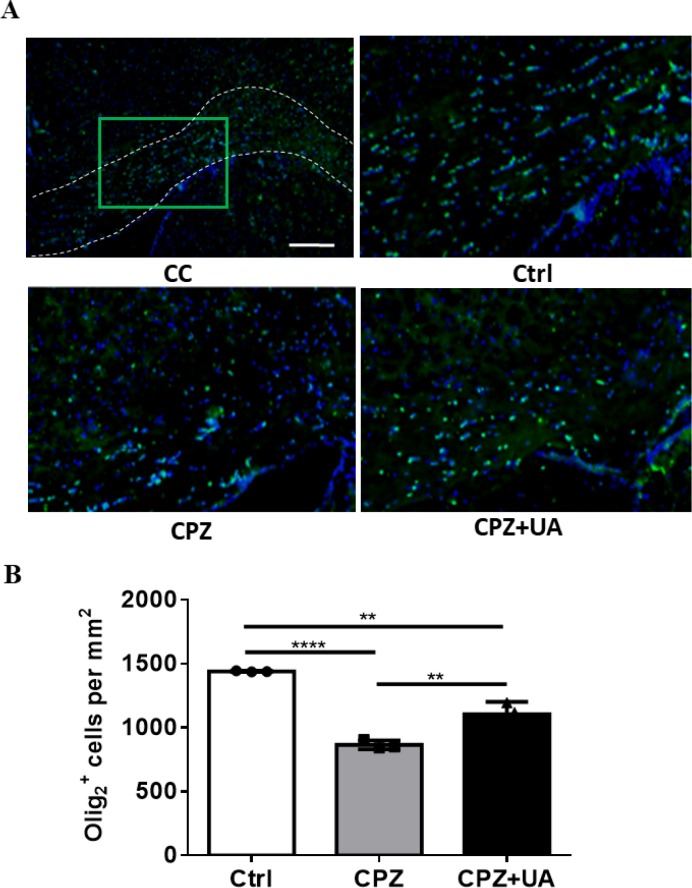
Changes in number of Olig2+ cells as a marker protein for oligodendrocyte lineage cells following 6 weeks CPZ feeding

To examine the preservative effect of UA in cellular level we performed immunostaining against Olig2 a marker of OL lineage cells (including both progenitors (OPCs) and mature OLs). The results demonstrated a reduced numbers of Olig2+ cells in CPZ-treated mice with implication of possible cell loss by CPZ toxicity as well as a preservation of OLs and/or OPCs by UA (Figure. 6A). Quantification of the number of Olig2+ cells showed that while the number of Olig2+ cells was reduced by CPZ (*p *< 0.001), UA administration in drinking water led to observing a higher number of OL lineage cells within the CC (*p *< 0.01) that suggests UA has the capability for preserving the oligodendrocyte lineage.

## Discussion

Ursolic acid is a secondary metabolite found in a variety of natural plants that has exhibited a wide range of pharmaceutical properties (24, 25). There is a report that UA could decrease diet-induced obesity. Kunkel *et al*. (26) indicated that UA as a natural substance in apple peel can partially protect mice from obesity and increased the skeletal muscle Akt activity. Up to our knowledge, our study is the first report that shows UA can protect myelin in CNS against toxin induced demyelination. This effect was investigated in the cuprizone induced demyelination model of C57/BL6 mice CNS. We hypothesized that UA may affect the appetite of mice during CPZ-feeding and reduce CPZ consumption in UA+CPZ group and checked the animals’ food intake. Our results demonstrated that the food consumption in the mice was not changed by UA during 6 weeks CPZ feeding. In addition, the drinking water consumption was not significantly changed in all the groups treated with and without UA. Therefore, we confirmed that UA could not reduce the consumption of CPZ food and the observed later changes in the myelination level can be attributed to UA preservative effect on myelin sheaths. As a supportive study, Xiang et al. (27) did not detect the weight loss side effect following UA administration in mice.

Many studies have demonstrated that UA could decrease or cease excitotoxicity and oxidative stress within the nervous system (20, 28-30), biological processes which widely contribute to myelin loss in multiple sclerosis patients symptoms as well as in animal models of MS. It also has had anti-inflammatory and neuroprotective effects. For example, UA could suppress the activation of immunoregulatory transcription factors like NF-κB, NF-AT, and AP-1 in lymphocytes (31). Within the inflamed central nervous system, immune system will be also activated mostly by increasing the number of activated microglia cells and astrocytes. It could reduce the interleukin and tumor necrosis factor and regulated the immune functions (32). All together, these reports support the possible protective effect of UA in neurodegenerative diseases including the multiple sclerosis as the most redundant neuro-inflammatory disease in young adults.

The first reported protective effect for UA on neural cells was indicated by a study that showed pretreatment with UA significantly decreased damage and suppressed free radical generation in kinate induced neurodegeneration in hippocampus (33). Wu *et al.* discovered intracellular signaling pathways involved in UA neuroprotection against cognitive impairment (34). Sahu *et al.* obtained an interesting insight into the neuroregeneration and regrowth potential of UA using the mouse model of spinal cord injury by mimicking human natural killer-1 (HNK-1) glycan (35). They demonstrated that UA decrease astrogliosis and promote the recovery of motor functions and axonal regrowth. In addition, UA suppressed pro-inflammatory cytokines such as IL-6 and TNF-α through signal transduction pathways including the mitogen-activated protein kinase (MAPK) and phosphoinositide 3-kinase (PI3K)/Akt/mammalian target of rapamycin (mTOR) pathways in the injured spinal cord. Here, we investigated the UA effect on CC demyelination in animal model of MS. We demonstrated that UA significantly maintains myelin in CC regions of CPZ-fed mice.

Although UA promoted the regeneration of injured sciatic nerve and increased the myelination following peripheral nerve injury (23), our study was the first report to show an effect for UA in which UA increased myelin and myelin survival following CNS demyelination ( focused on CC). To exhibit the protective functions of UA on CNS myelin, we performed the techniques such as myelin specific staining using FM and LFB as well as immunohistofluorescence study against mature myelin marker MBP to determine the demyelination rate. In the first step, we determined the presence of myelin loss in animal groups treated with CPZ. Then, we showed that UA in drinking water preserved the myelin when compared to CPZ group. Via calculating the extent of demyelination area or myelination intensity within CC. UA indicated a protection by lower amounts in the extent of myelinated area or higher myelin stain intensity.

In addition, UA-treated mice showed a significantly increased number of OL lineage cells stained with Olig2 antibody. Olig2 is expressed in both OPCs and mature oligodendrocytes and measuring the number of Olig2+ cells give us a general estimation on the population of cells that myelinate or differentiate into myelinating cells. Therefore, we suggested that UA reduced demyelination via protecting the related cells following CPZ administration. However, the mechanisms of myelin protection remain to be clarified. The higher number of Olig2+ cells may be also due to generation of new Olig2+ cells and therefore enhanced repair, a possible mechanism which need to be studied in future works. 

OLs generation is a multiple-step process that is included the proliferation, migration, and differentiation of oligodendroglial progenitor cells into mature oligodendrocytes (36). The number of olig2 + cells were significantly reduced in CPZ group which imply OLs loss. 

The impact of UA on the brain is not limited to the cellular level. Xu and colleagues (37) showed that behavioral and neurobiological changes occurred in C57BL/6 mice exposed to cuprizone. To confirm the presence of behavioral impairment due to demyelination and extensive demyelination in the brains of the mice, we conducted the Y-maze test and evaluated the influence of UA on mice’s behavior. Our findings demonstrated that the UA-treated mice showed an improved behavior in Y-maze with reduced memory impairment.

Therefore, our results demonstrate that UA prevents demyelination in corpus callosum when administrated during the course of demyelination induction with cuprizone. We suggest that UA could activate a neuroprotective strategy and improve the myelin and myelinating cells survival in demyelinated corpus callosum axons in cuprizone model of MS.

(B) Quantitative analysis of the demyelination intensity in LFB-stained sections. The extent of demyelination was normalized to the total area of CC in one-half of brain. Ctrl: animals, which received normal food and water, CPZ: animals, which received normal water and cuprizone food, CPZ+UA: animals, which received cuprizone food and UA in drinking, water. **p < 0.01, compared to CPZ group, *p < 0.05 and**** *p *< 0.0001 compared to the control group. Scale bars: 100 µm, n = 3.

(A) Immunofluorescence images of oligodendrocyte lineage cells (OPCs+ OLs) stained using Olig2 antibody. (B) Quantification of Olig2+ cells as counted number per area (mm2). Cells were counterstained with DAPI. ***p < 0.001 compared to CPZ group, **p <0.01 compared to CPZ+UA group. Scale bars: 100 μm. N = 3.

**Table 1:** List of primary and secondary antibodies used in this study

**Antigen**	**Species**	**Catalog#**	**Condition**	**Label**
**Olig2**	Rabbit polyclonal IgG	Abcam, Inc. ab9610	1:200	-
**MBP**	Chicken polyclonal anti-peptide antibody mixture	Aves	1:2000	-
**Rabbit IgG**	Goat anti-rabbit	Life Technologies, A11036	1:1000	Alexa Fluor® 488
**Chicken IgG**	Rabbit anti-chicken	Abcam, Inc. ab6751	1:500	Texas Red

## Conclusion

In the current study, we showed that the groups of animals which received daily treatment UA in drinking water, when simultaneously were challenged with a demyelinating insult by CPZ, could protect the myelin and the OL lineage cells. The results obtained in the present study indicate that UA therapy protected myelination as measured by FM and LFB staining and MBP staining. Although the molecular mechanisms responsible for the therapeutic effects of UA on demyelination remain unclear, we suggest that UA may modulate neuroimmune and inflammatory processes and provide a favorable external environment for neural cells and their protection. 
